# Heterogeneity of neuroblastoma

**DOI:** 10.18632/oncoscience.216

**Published:** 2015-08-24

**Authors:** Elly Sau-Wai Ngan

**Affiliations:** Department of Surgery, Li Ka Shing Faculty of Medicine, University of Hong Kong, Pokfulam, Hong Kong

**Keywords:** neuroblastoma, heterogeneity, tumor initialing cells

Neuroblastoma (NB) is the most common pediatric solid tumor and represents 15% of all childhood cancer deaths. High cellular heterogeneity is a hallmark of NB, which may account for the wide range of clinical presentations and non-uniform response to treatment. Compelling evidence indicates that cancer cells in solid tumors may not necessarily be hierarchically organized, but rather, also following the clonal evolution model in which cancer cells may evolve from one subtype to the others, generating tumors with distinct features. Cancer cells may even transiently acquire stemness properties depending on the tumor context [1–3]. Importantly, these two models are not mutually exclusive in cancers, accounting for the tumor heterogeneity [4].

To date, there is still no defined surface marker available for the identification of tumor initiating cells (TICs) from NB. TICs isolated from the high staged NBs were mainly based on their stem cell characteristics, including sphere-formation capacity and plasticity with respect to differentiation along several lineages [5, 6]. Transcriptome analysis of NB TICs further highlighted the complexity of these cells and suggested that NB TICs may exist as a dynamic and heterogeneous cell population strongly dependent on the particular niche environment [5].

In our recent manuscript by Liu et. al. [7], we made use of various lines of NB-TICs isolated from the primary and bone-marrow metastasized NB, and NB cell lines to generate *in vivo* models for studying tumor heterogeneity. We discovered that a population of NB cells, which express high level of c-KIT (c-KIT^high^), a tyrosine-kinase receptor for stem cell factor (SCF), is *de novo* generated and dynamically maintained within the tumors to sustain tumor progression. c-KIT^high^ NB cells possess the stem cell characteristics: they expressed high levels of neural crest and stem cell markers (*SLUG*, *SOX2*, *NANOG*), possess high clonogenic capacity, differentiation plasticity and drug resistance. Even though c-KIT expression is not required for the tumor formation, serial transplantation assays showed that c-KIT^high^ cells are more aggressive and induce tumors 9-fold more efficiently than c-KIT^−/low^ cells. More importantly, c-KIT^high^ cells exhibited a long-term *in vivo* self-renewal capacity to sustain the formation of the secondary and tertiary tumors in mice. All these data support the notion that NB cells change dynamically, they evolve and generate new progenies during tumor progression. The progenies would be more tumorigenic than the parental cells to sustain the tumor progression. Since c-KIT^−/low^ cells can give rise to c-KIT^high^ cells and *vice versa*, targeting c-KIT may not be sufficient to effectively eradicate the tumor. Thus, we proposed to target a growth factor, namely Prokineticin, where its receptors are ubiquitously expressed in both c-KIT^high^ and c-KIT^−/low^ cells and supports the formation and growth of c-KIT^high^ cells. We showed that knocking down the receptors for Prokineticin provides a more effective way to inhibit the tumor formation than directly targeting c-KIT, by abolishing the *de novo* generation of c-KIT^high^ cells. In summary, we have employed NB-TIC lines isolated from primary and bone-marrow metastasized tumors to establish an *in vivo* cancer model for understanding how NB TICs give rise to heterogeneous derivatives and how these derivatives are organized and influence disease progression or treatment response. We also provided evidence that targeting the growth factor which supports the formation of moving target represents an effective way to eradicate NB.

Obviously, NB is highly heterogeneous, comprising cancer cells with very different molecular features. The next important questions are to understand how the clonal composition of a tumor influences its progression and treatment response, how the molecular features determine the behavior of tumor cells, and what are the common features or molecular signatures shared by the most aggressive tumor cells of various tumors. NB-TIC derived tumor model would be a useful platform for understanding tumor formation, progression and tumor heterogeneity. Recent advances in single cell sequencing technology allow a precise examination of the expression profile of the heterogeneous cancer populations at a single cell level. Comparisons of the molecular signatures of the tumor cells derived from various NB-TICs will allow a systematic analysis of heterogeneous populations of a tumor and help identify candidate genes for future drug intervention. Nevertheless, the most formidable challenge of the field is to know which population of cancer cells within the individual tumor we should target: TIC or its most aggressive progenies, in order to translate our knowledge of cancer biology to establish the personalized treatment strategy for the patients.
